# Diagnostic accuracy of semi-quantitative and quantitative culture techniques for the diagnosis of catheter-related infections in newborns and molecular typing of isolated microorganisms

**DOI:** 10.1186/1471-2334-14-283

**Published:** 2014-05-22

**Authors:** Danilo Flávio Moraes Riboli, João César Lyra, Eliane Pessoa Silva, Luisa Leite Valadão, Maria Regina Bentlin, José Eduardo Corrente, Ligia Maria Suppo de Souza Rugolo, Maria de Lourdes Ribeiro de Souza da Cunha

**Affiliations:** 1 Departamento de Microbiologia e Imunologia, Instituto de Biociências, UNESP - Univ Estadual Paulista, Botucatu, SP, Brasil; 2 Departamento de Pediatria, Faculdade de Medicina, UNESP - Univ Estadual Paulista, Botucatu, SP, Brasil; 3 Departamento de Bioestatística, Instituto de Biociências, UNESP - Univ Estadual Paulista, Botucatu, SP, Brasil

**Keywords:** Semiquantitative culture, Quantitative culture, Catheter-related bloodstream infections, Sensitivity and specificity

## Abstract

**Background:**

Catheter-related bloodstream infections (CR-BSIs) have become the most common cause of healthcare-associated bloodstream infections in neonatal intensive care units (ICUs). Microbiological evidence implicating catheters as the source of bloodstream infection is necessary to establish the diagnosis of CR-BSIs. Semi-quantitative culture is used to determine the presence of microorganisms on the external catheter surface, whereas quantitative culture also isolates microorganisms present inside the catheter. The main objective of this study was to determine the sensitivity and specificity of these two techniques for the diagnosis of CR-BSIs in newborns from a neonatal ICU. In addition, PFGE was used for similarity analysis of the microorganisms isolated from catheters and blood cultures.

**Methods:**

Semi-quantitative and quantitative methods were used for the culture of catheter tips obtained from newborns. Strains isolated from catheter tips and blood cultures which exhibited the same antimicrobial susceptibility profile were included in the study as positive cases of CR-BSI. PFGE of the microorganisms isolated from catheters and blood cultures was performed for similarity analysis and detection of clones in the ICU.

**Results:**

A total of 584 catheter tips from 399 patients seen between November 2005 and June 2012 were analyzed. Twenty-nine cases of CR-BSI were confirmed. Coagulase-negative staphylococci (CoNS) were the most frequently isolated microorganisms, including *S. epidermidis* as the most prevalent species (65.5%), followed by *S. haemolyticus* (10.3%), yeasts (10.3%), *K. pneumoniae* (6.9%), *S. aureus* (3.4%), and *E. coli* (3.4%). The sensitivity of the semi-quantitative and quantitative techniques was 72.7% and 59.3%, respectively, and specificity was 95.7% and 94.4%. The diagnosis of CR-BSIs based on PFGE analysis of similarity between strains isolated from catheter tips and blood cultures showed 82.6% sensitivity and 100% specificity.

**Conclusion:**

The semi-quantitative culture method showed higher sensitivity and specificity for the diagnosis of CR-BSIs in newborns when compared to the quantitative technique. In addition, this method is easier to perform and shows better agreement with the gold standard, and should therefore be recommended for routine clinical laboratory use. PFGE may contribute to the control of CR-BSIs by identifying clusters of microorganisms in neonatal ICUs, providing a means of determining potential cross-infection between patients.

## Background

Catheter-related bloodstream infections (CR-BSIs) have become the most common cause of healthcare-associated bloodstream infections and are associated with substantial morbidity and mortality and excessive hospital costs [1,2]. In Brazil, CR-BSIs are the leading infection in neonatal intensive care units (ICUs) [3].

The diagnosis of CR-BSIs continues to be a challenge. Fever and chills are common signs of infection, but are not specific, and the sensitivity of inflammation at the site of catheter insertion is 8% or less [4]. Therefore, microbiological evidence implicating the catheter as a source of bloodstream infection is necessary to establish the diagnosis of CR-BSIs.

In view of the lack of a gold standard, the microbiological criterion is a subject of intense research and its clinical relevance is frequently discussed by experts. The semi-quantitative method proposed by Maki et al. in 1977 [5] continues to be the international reference diagnostic method and is used as the gold standard in studies comparing different diagnostic methods. In this culture method, a segment of the catheter tip is rolled across a blood agar plate to detect the presence of bacteria on the external catheter surface. A count of 15 colony-forming units (CFU) or more on the plate indicates colonization of the catheter [5,6]. Subsequently, an endoluminal culture system was developed by Cleri et al. in 1980 [7] to avoid the loss of intraluminal microorganisms. A modification of the procedure was proposed by Brun-Buisson et al. in 1987 [8], which consisted of diluting the bacteria in sterile water followed by vortexing for determination of the presence of microorganisms on the external and internal surface of the catheter. The threshold for a significant count is 10^3^ CFU/mL [7,8].

According to the CDC [6], an infection can only be defined as catheter related if, in addition to a result of ≥ 15 CFU in semi-quantitative culture of the catheter tip or of 10^3^ CFU/mL in quantitative culture, the same microorganism is isolated from a peripheral blood culture and there are clinical signs of sepsis. For the elucidation of CR-BSI, it is fundamental to determine similarities between microbial strains isolated from catheter tips and blood cultures. Biochemical tests are helpful to determine the genus and species of the microorganism and antibiotic susceptibility testing is used to evaluate similarities between strains isolated from catheters and blood cultures based on their resistance profile. These approaches contribute not only to the treatment of CR-BSIs, but also to the diagnosis of these infections.

In an attempt to develop more specific and sensitive methods for the diagnosis of CR-BSIs that are able to establish genetic relationships between isolates obtained from catheters and blood cultures, molecular typing techniques may provide an additional discriminatory potential, especially in the case of infections in which the pathogen is part of the normal microbiota. The understanding of the relationship between microorganisms is fundamental for the elucidation of CR-BSIs. Pulsed-field gel electrophoresis (PFGE) is a molecular typing method that is based on the digestion of DNA with restriction enzymes which generates different genotype profiles after pulsed-field electrophoresis. This method is the gold standard for the typing of microorganisms involved in hospital outbreaks and in surveillance studies to detect the presence of circulating clones [9].

Since the semi-quantitative culture method only detects the presence of microorganisms on the external catheter surface, whereas quantitative culture also isolates microorganisms present inside the catheter, the main objective of this study was to determine the accuracy of the semi-quantitative technique proposed by Maki et al. [5] and of the quantitative technique described by Brun-Buisson et al. [8] for the diagnosis of CR-BSIs in newborns from a neonatal ICU. In addition, PFGE was used for similarity analysis of the microorganisms isolated from catheters and blood cultures.

## Methods

The microorganisms studied were isolated from catheter tips and blood cultures of newborns hospitalized in the neonatal ICU of the University Hospital of the Botucatu Medical School (Hospital das Clínicas, Faculdade de Medicina de Botucatu-HC-FMB) between November 2005 and June 2012.

The University Hospital of FMB is a tertiary care hospital. The neonatal ICU currently has 17 beds and offers high-complexity service, attending mainly preterm infants weighing less than 1500 g. The maternity unit is a referral center for high-risk pregnancies and receives patients born at the hospital and also from other secondary services in the region.

The present study was approved by Committee for Ethics in Research (“Comitê de Ética em Pesquisa” from “Faculdade de Medicina de Botucatu”, Botucatu, São Paulo State, Brazil-Protocol 3522–2010).

All catheter tips showing growth by at least one of the culture techniques and with at least one peripheral blood culture collected from newborns 7 days before or after removal of the catheter were included in the study. Excluded were catheter tips of newborns for whom no clinical and laboratory records comprising the period between one week before and one week after the date of device removal were available.

CR-BSI was confirmed when the inclusion criteria were met, when the same microorganism and the same species were identified, and when the microorganisms isolated from peripheral blood cultures and catheter tips exhibiting growth ≥ 15 CFU by the semi-quantitative technique of Maki et al. [5] and/or ≥ 1,000 CFU/mL by the quantitative technique of Brun-Buisson et al. [8] had the same antimicrobial susceptibility profile. In addition, the newborn should present at least one of the following signs and symptoms: thermal instability, bradycardia, apnea, food intolerance, worsening of respiratory distress, glucose intolerance, hemodynamic instability, and hypoactivity/lethargy in the absence of any other recognized non-infectious cause and of any relationship with infection at another site [10].

Collection and culture of the catheter tips, as well as the collection of clinical data, were done prospectively; however, some clinical data were obtained retrospectively from the medical records.

Catheter tips were cultured using the semi-quantitative method proposed by Maki et al. [5] and the quantitative method proposed by Brun-Buisson et al. [8]. The catheters were removed aseptically by the medical team of the hospital and two tips measuring approximately 5 cm were transferred to a sterile dry tube and transported immediately to the laboratory for processing. For the semi-quantitative technique, distal segments were rolled across the surface of a blood agar plate and incubated for 72 h at 37°C. The plates were examined daily and colonies were counted as soon as growth was observed. The results are expressed as CFU. Proximal segments of the catheter tip were cultured using a quantitative method. For this purpose, the catheter was flushed with 1 mL sterile distilled water, the solution was vortexed for 1 min, and an aliquot of 0.1 mL was spread on a blood agar plate with a Drigalski spatula. The plates were incubated for 72 h at 37°C, examined daily, and colonies were counted as soon as growth was observed. The results are expressed as CFU.

The catheter tip cultures were always prepared by the same previously trained researcher. The findings were discussed with the coordinator of the study and the results of the two techniques were analyzed concomitantly. Blood was collected and cultured using the automated Bactec system according to the recommendations of Koneman et al. [11].

After the observation of growth on the blood agar plate, the microorganisms were submitted to Gram staining for the analysis of purity of the strain, morphology, and specific staining. The microorganisms were identified as described by Koneman et al. [11]. Gram-positive cocci were submitted to the catalase test for differentiation of the genera *Staphylococcus* and *Streptococcus*. The coagulase test was used for the identification of *Staphylococcus aureus*. CoNS were identified using a simplified scheme of biochemical tests as proposed by Cunha et al. [12], including the utilization of xylose, sucrose, trehalose, mannitol and maltose, anaerobic growth on thioglycolate, nitrate reduction, and presence of urease and ornithine decarboxylase. Gram-negative bacilli were submitted to manual biochemical tests, including glucose fermentation, gas release, production of H_2_S, urease, L-tryptophane deaminase, motility test, indol, production of lysine decarboxylase, and growth on citrate.

Antimicrobial susceptibility of the microbial isolates was tested by the agar disk diffusion method according to the criteria of the Clinical Laboratory Standards Institute (2013). Disks impregnated with the following drugs were used: penicillin (10 μg), oxacillin (1 μg), cefoxitin (30 μg), erythromycin (15 μg), cephalothin (30 μg), gentamicin (30 μg), and rifampicin (30 μg). The susceptibility profile of yeasts was tested using amphotericin B (100 μg) and fluconazole (25 μg) disks [13].

PFGE of *Staphylococcus* spp. and *Klebsiella pneumoniae* strains isolated from catheters and blood cultures was performed according to protocols modified from McDougal et al. [14] and Durmaz et al. [15], respectively.

For restriction of genomic DNA, 2 μL of *SmaI* (Fast Digest SmaI, Fermentas Life Science, Canada) for *Staphylococcus* spp. and of *XbaI* for *Klebsiella pneumoniae* were used. Electrophoresis was performed in a CHEF-DR III System (BioRad Laboratories, USA) using 1% agarose gel (Pulsed Field Certified Agarose, BioRad Laboratories, USA) prepared in 0.5 × TBE under the following conditions: pulse time interval of 5 to 40 s for 21 h for *Staphylococcus* spp. and 5 to 90 s for 23 h for *Klebsiella pneumoniae*; linear ramp; 6 V/cm; angle of 120°; 14°C; 2.2 L of 0.5 × TBE as running buffer. The Lambda Ladder PFG Marker (New England BioLabs, Canada) was used as molecular weight marker. The gels were stained with GelRed (400 mL distilled water and 30 μL GelRed 10,000X in water; Biotium, USA) for 1 h and photographed under UV transillumination.

The BioNumerics software (version 6.1; Applied Maths, Belgium) was used for similarity analysis, calculation of the Dice correlation coefficient, and generation of a dendrogram by the UPGMA method. Band position tolerance and optimization were adjusted to 1.25 and 1%, respectively. A similarity coefficient of 80% was chosen for cluster definition [14].

The accuracy of the culture methods for the diagnosis of CR-BSIs was determined by calculating sensitivity and specificity. The kappa coefficient was used to evaluate agreement between the culture methods and the gold standard using a 95% confidence interval. The gold standard consisted of the definitive diagnosis of CR-BSI by isolation of the same microorganism (species and antibiotic susceptibility profile) from catheter and blood cultures and the diagnosis of sepsis according to the clinical criteria of the unit [10]. For determination of the accuracy of the techniques, newborns with more than one episode of CR-BSI were excluded from the analysis.

## Results

### Sample collection

**Figure 1:**
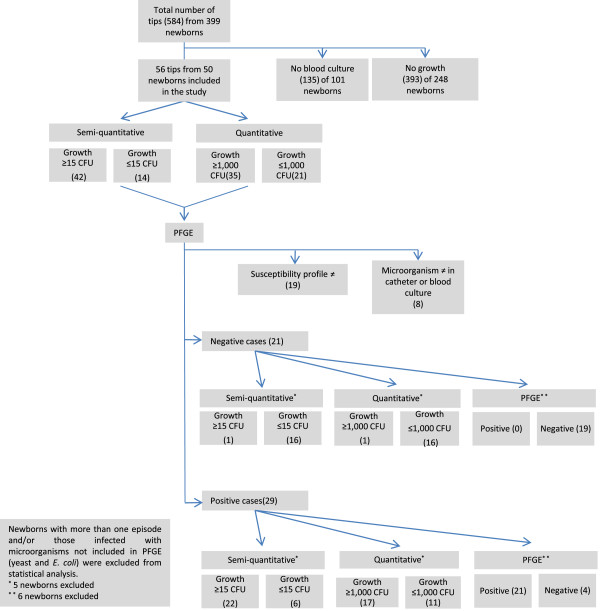
Flow diagram of the diagnosis of catheter-related infections in newborns.

A total of 584 catheter tips from 399 newborns, hospitalized in the neonatal ICU of HC-FMB between November 2005 and June 2012, were analyzed. Of these, 56 catheter tips from 50 newborns were included in the study according to the inclusion criteria. Twenty-nine of the 50 newborns were classified as having CR-BSI according to the gold standard, i.e., isolation of the same microorganism (the same species and antimicrobial susceptibility profile) from catheter and blood cultures and diagnosis of sepsis according to clinical criteria (Figure 1).

Table 1 shows the personal and clinical data of the newborns included in the study. Most newborns were preterm, had a low birth weight, and had received parenteral nutrition, mechanical ventilation and a short-term catheter (up to 10 days).

**Table 1:** Personal and clinical data of the newborns included in the study (n = 50)

		**n**	**%**
Gestational age (weeks)	< 31	30	60
	31-36	11	22
	> 36	9	18
Birthweight (g)	< 1,000	24	48
	1.000-1.499	9	18
	1.500-2.499	4	8
	> 2.500	13	26
Gender	Male	28	56
	Female	22	44
Catheter duration	≤ 10 days	38	76
	> 10 days	12	24
Type of catheter	Umbilical vein	15	30
	Umbilical artery	9	18
	PICC*	26	52
Parenteral nutrition		40	80
Mechanical ventilation		38	76
Death	Yes	15	30
	No	35	70

*Peripherally inserted central catheter.

### Culture of catheter tips

Forty-two of the 56 catheter tips analyzed exhibited growth ≥ 15 CFU, indicating colonization of the catheter according to the criteria proposed by Maki et al. [5], and 35 exhibited growth ≥ 1,000 CFU/mL according to the quantitative technique of Brun-Buisson et al. [8] (Table 2).

**Table 2:** Diagnosis of catheter-related bloodstream infection by the semi-quantitative and quantitative techniques

	**Semi-quantitative technique**				**Quantitative technique**			
	**≥ 15 CFU**		**≤ 15 CFU**		**≥ 1,000 CFU**		**≤ 1,000 CFU**	
	**n**	**%**	**n**	**%**	**n**	**%**	**n**	**%**
**Positive catheters (n = 56)**	42	75.0	14	25.0	35	62.5	21	37.5
**CR-BSI (n = 29)**	23	79.3	6	26.0	18	54.5	11	37.9

CR-BSI: catheter-related bloodstream infection.

Twenty-three (79.3%) of the 29 cases of CR-BSI tested positive by the semi-quantitative technique, with growth ≥ 15 CFU, and 18 (62.1%) exhibited growth ≥ 1,000 CFU by the quantitative technique. In four cases, growth was ≥ 15 CFU in semi-quantitative culture, but < 1,000 CFU in quantitative culture. There was one case in which no growth was observed in the quantitative culture. Comparison of the semi-quantitative technique with the gold standard revealed five cases of growth < 15 CFU and one case in which no growth was observed in the semi-quantitative culture. Of these six cases, three did not grow in quantitative culture and the three other cases exhibited growth < 1,000 CFU. Therefore, all cases of CR-BSI diagnosed by quantitative culture were also positive by semi-quantitative culture.

CoNS were the most common microorganisms (n = 17, 73.9%) detected by the semi-quantitative technique. *S. epidermidis* was isolated from 14 (61%) of the 23 cases of CR-BSI and *S. haemolyticus* from 3 (13%). *S. aureus* was isolated from only one case of CR-BSI (4.3%). Gram-negative bacteria were isolated from 3 (13%) cases of CR-BSI, including *K. pneumoniae* from 2 (8.7%) and *E. coli* from one (4.3%). Yeasts were isolated from 2 (8.7%) cases (Table 3).

**Table 3:** Incidence of microorganisms associated with catheter-related bloodstream infections detected by semi-quantitative and quantitative culture

	**Semi-quantitative culture**		**Quantitative culture**	
**Microorganism**	**≥ 15 CFU**	**< 15 CFU**	**≥ 1,000 CFU**	**< 1,000 CFU**
***S. epidermidis *** **(n = 19)**	14	5^*^	10	9^**^
***S. haemolyticus *** **(n = 3)**	3	0	3	0
***S. aureus *** **(n = 1)**	1	0	1	0
***Klebsiella pneumoniae *** **(n = 2)**	2	0	2	0
**Yeast (n = 3)**	2	1	1	2^*^
***E. coli *** **(n = 1)**	1	0	1	0
**Total (n = 29)**	23	6	18	11

^*^One culture without growth; ^**^Three cultures without growth.

Semi-quantitative culture did not detect growth in one case of CR-BSI caused by *S. epidermidis* and growth below the cut-off (< 15 CFU) was observed in five cases, four caused by *S. epidermidis* and one by yeast (Table 2). No growth of microorganisms was detected by the quantitative technique in four episodes, including three caused by *S. epidermidis* and one by yeast. Growth in quantitative culture below the cut-off (< 1,000 CFU) was observed in six episodes caused by *S. epidermidis* and in one caused by yeast (Table 3).

Only 25 of the 29 cases of CR-BSI were submitted to PFGE since *E. coli* and yeasts were not analyzed by this technique. PFGE confirmed the similarity of microorganisms isolated from catheter and blood cultures in 21 (84%) of the 25 cases analyzed. Considering the 23 cases of CR-BSI diagnosed by the semi-quantitative technique and excluding the two episodes caused by yeast and one episode caused by *E. coli* that were not submitted to molecular typing by PFGE, confirmation was obtained in 18 (90%) of 20 cases.

**Figure 2:**
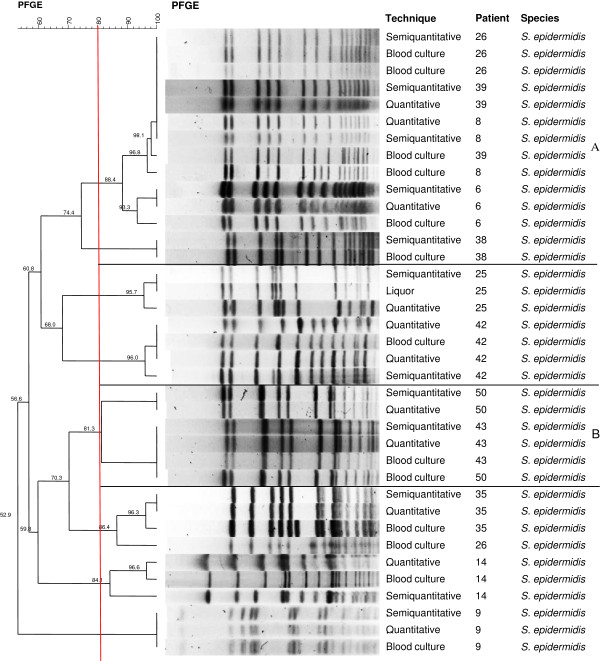
**Dendrogram generated by Dice/UPGMA analysis (Bionumerics, Applied Maths) of *SmaI *** **PFGE profiles of *S. epidermidis *** **isolated from catheters by semi-quantitative and quantitative culture and from blood cultures of confirmed cases of catheter-related bloodstream infection (similarity ≥ 80%).**

PFGE confirmed 12 of the 14 cases caused by *S. epidermidis* (patients 6, 8, 9, 14, 25, 26, 35, 38, 39, 42, 43, and 50) (Figure 2). Among these cases, there was a predominant cluster (group A) which exhibited similarity > 80% in four cases of CR-BSI, including patients 6, 8, 26 and 39 from which the same strain was isolated in 2006, 2007, 2009 and 2010, respectively. Analysis of the dendrogram also revealed similarity > 80% between microorganisms isolated from patients 43 and 50 (group B) in 2011 and 2012, respectively.

**Figure 3:**
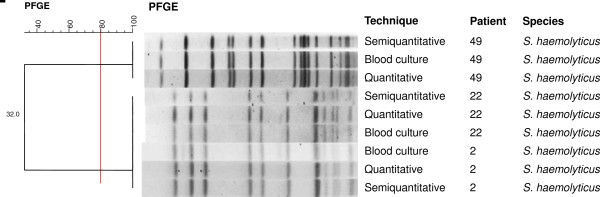
**Dendrogram generated by Dice/UPGMA analysis (Bionumerics, Applied Maths) of *SmaI *** **PFGE profiles of *S. haemolyticus *** **isolated from catheters by semi-quantitative and quantitative culture and from blood cultures of confirmed cases of catheter-related bloodstream infection (similarity ≥ 80%).**

The three cases of CR-BSI caused by *S. haemolyticus* were also confirmed by PFGE (patients 2, 22, and 49) (Figure 3). Two catheter tips from patient 22 obtained on different days (interval of 2 days) were cultured and both showed confluent growth by the two methods. However, the same microorganism was isolated from blood culture in only one case. The microorganisms isolated from patients 2 and 22 in 2006 and 2008, respectively, showed 100% similarity.

**Figure 4:**
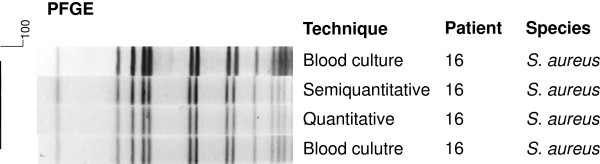
**Dendrogram generated by Dice/UPGMA analysis (Bionumerics, Applied Maths) of *SmaI *** **PFGE profiles of *S. aureus *** **isolated from catheters by semi-quantitative and quantitative culture and from blood cultures of newborns included in the study (similarity ≥ 80%).**

The only confirmed case of CR-BSI caused by *S. aureus* was also confirmed by PFGE, showing 100% similarity (Figure 4).

**Figure 5:**
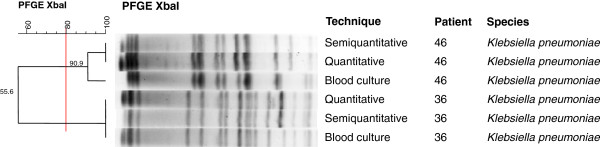
**Dendrogram generated by Dice/UPGMA analysis (Bionumerics, Applied Maths) of *XbaI *** **PFGE profiles of *K. pneumoniae *** **isolated from catheters by semi-quantitative and quantitative culture and from blood cultures of newborns included in the study (similarity ≥ 80%).**

The two cases of infection caused by *K. pneumoniae* were confirmed by PFGE (100% and 90.9% similarity). However, analysis of the dendrogram revealed two distinct clones (Figure 5).

The sensitivity of the semi-quantitative and quantitative methods was 72.7% and 59.28%, respectively, and specificity was 95.7% and 94.4% (see Additional file 1). The diagnosis of CR-BSIs based on the similarity of isolates from catheter tips and blood cultures determined by PFGE genotyping showed a sensitivity of 82.6% and specificity of 100%. The kappa coefficient of agreement between the techniques and the gold standard was 0.6872 (substantial agreement) for the semi-quantitative technique, 0.4915 (moderate agreement) for the quantitative technique, and 0.8193 (excellent agreement) for PFGE (Table 4).

**Table 4:** Sensitivity and specificity of the techniques analyzed and kappa coefficient

**Technique**	**Sensitivity**	**Specificity**	**Kappa**	**95% CI**	
				**Lower**	**Upper**
**Semi-quantitative**	72.7	95.7	0.6872	0.4797	0.8947
**Quantitative**	59.2	94.4	0.4915	0.2677	0.7154
**PFGE**	82.6	100	0.8193	0.6532	0.9854

## Discussion

There are several comparative studies of semi-quantitative and quantitative culture of catheter tips for the diagnosis of CR-BSIs [16,17]. However, these studies always involved adult patients. We conducted this study in view of the lack of specific studies in the literature comparing these culture methods to improve the diagnosis of CR-BSIs in newborns.

In the present study, the semi-quantitative technique identified more cases of CR-BSI (n = 23) than the quantitative culture method (n = 18). Using quantitative culture, growth below the cut-off (< 10^3^ CFU/mL) was observed in one case caused by yeast and in three cases caused by *S. epidermidis*. No growth was observed in one case associated with *S. epidermidis*. In contrast, growth > 15 CFU of these five isolates was detected by the semi-quantitative culture method. These results may be explained by the contamination with microorganisms adhered only to the external surface of the device and by the fact that short-term catheters (up to 10 days) were used in most cases. Contamination of the external catheter surface has been reported as the main route of infection of short-term catheters, whereas intraluminal contamination is an important route of infection in long-term catheterization [18]. In addition, the density of colonizing bacteria is higher on the external than on the internal surface of the catheter [6,19].

The sensitivity of the semi-quantitative and quantitative techniques was 72.7% and 59.28%, respectively, and specificity was 95.7% and 94.4%. There were six episodes of CR-BSI that were not detected by semi-quantitative or quantitative culture, five of them associated with *S. epidermidis* and one with yeast. One limitation of the semi-quantitative culture method is that it only detects colonization of the external catheter surface and not intraluminal colonization. Furthermore, the lower sensitivity of semi-quantitative and, particularly of quantitative culture, observed in the present study may be explained by the fact that antibiotic therapy is frequently administered to preterm newborns in ICUs. This treatment reduces the yield of bacterial culture of both the external and internal surface of catheters. Since antibiotics are administered through a central venous catheter, microorganisms present inside the catheter are exposed to higher concentrations of the antibiotic than bacteria found on the external surface. Therefore, previous administration of antibiotics can influence the sensitivity of detection methods, particularly quantitative culture.

In a meta-analysis comparing six culture techniques, Siegman-Igra et al. [20] reported the superiority of quantitative culture methods of catheter segments. The authors concluded that quantitative culture of catheter segments shows greater precision in the diagnosis of CR-BSIs and is the only method with sensitivity and specificity higher than 90% and the best cost-benefit relationship [20]. However, according to Bouza et al. [17], quantitative techniques are not superior to the method of Maki et al. [5] when used under the same conditions. Therefore, the sensitivity, specificity and simplicity of the semi-quantitative technique proposed by Maki et al. [5] makes it the method of choice for routine use in clinical microbiology laboratories.

According to the National Nosocomial Infections Surveillance (NNIS) [21], CoNS were responsible for 37.7% of bloodstream infections in pediatric ICUs between 1992 and 1999. More recent data reported by the National Healthcare Safety Network (NHSN) of the CDC also show that CoNS rank first place as the etiological agent of CR-BSIs (34.1%), followed by *Enterococcus* spp. (16%), *Candida* spp. (11.8%) and *S. aureus* (9.9%) in fourth place. Gram-negative microorganisms including enterobacteria such as *E. coli*, *Enterobacter*, *Klebsiella pneumoniae* and *Klebsiella oxytoca* were responsible for 12.4% of these infections [22]. In the present study, the only case of CR-BSI caused by *E. coli* resulted in the death of the patient in 2009. Although rare, CR-BSIs caused by these etiological agents are severe and are associated with a high rate of lethality (40-90%) since non-maternal strains of *E. coli* (from another patient or nosocomial) can cause severe invasive disease [23]. Considering the immature defense system of preterm newborns, the impact of infections caused by these microorganisms is even greater.

In addition to their virulence and ability to acquire antibiotic resistance, *Klebsiella* and *E. coli* are able to survive on the skin and moist surfaces, withstanding drying, and are easily transferred through equipment and the hands of the medical team [24]. Neonatal ICUs are the ideal place for the dissemination of these microorganisms as a result of extended hospital stay and prolonged use of antimicrobial agents, as well as the use of invasive devices [25].

The higher frequency of *S. epidermidis* in CR-BSIs is expected since it is the predominant species in the microbiota of newborns. This predominant colonization and the higher pathogenicity of some strains may explain the fact that *S. epidermidis* is the most common species associated with infectious processes in newborns as demonstrated by Cunha et al. [26,27]. The presence of clusters of *S. epidermidis*, with the isolation of strains from different newborns over a period of 4 years (2006 to 2010), and of *S. haemolyticus* (2006 to 2008) indicates the existence of prevalent clones of these CoNS species in neonatal ICUs. These microorganisms can colonize catheters and invade the bloodstream, causing systemic infection in this group of patients submitted to invasive procedures since most of them are preterm and low-birth weight newborns whose immature and deficient immune system contributes to infection. The presence of *S. haemolyticus* clones highlights the importance for control measures of CR-BSIs since this species has been described to be the most resistant to different antimicrobial agents and is an important carrier of resistance genes that can be transferred to other CoNS species causing CR-BSIs [28,29].

## Conclusion

The semi-quantitative culture technique showed higher sensitivity and specificity in the diagnosis of CR-BSIs in newborns when compared to quantitative culture. In addition, this method is simpler and shows better agreement with the gold standard and should therefore be recommended for routine use in clinical laboratories. PFGE permits to confirm whether the strain isolated from the catheter and blood of the patient is the same and may contribute to the control of CR-BSIs by identifying clusters of microorganisms in neonatal ICUs, providing a means of determining potential cross-infection between patients.

## Competing interests

The authors declare that they have no competing interests.

## Authors’ contributions

DFMR: participated in the conception and design of the study, carried out the microbiological tests, and wrote the paper. EPS: participated in the conception and design of the study, carried out the microbiological tests, analyzed the clinical data, and participated in the writing of the paper. LLV: helped with the microbiological tests. JCL (physician of the neonatal ICU): provided the clinical material and helped with the collection of clinical data. MRB (physician of the neonatal ICU): provided the clinical material and helped with the collection of clinical data. JEC (statistics consultant of the Botucatu Medical School): performed the statistical analysis of the results. LMSSR (head of the neonatal ICU): coordinated the collection of clinical material and analysis of clinical data. MLRSC: responsible for the conception and design of the study, coordination of laboratory work, data analysis, and manuscript writing. All authors read and approved the final manuscript.

## Pre-publication history

The pre-publication history for this paper can be accessed here:

http://www.biomedcentral.com/1471-2334/14/283/prepub

## Supplementary Material

Additional file 1: Table S1 Determination of the sensitivity and specificity of the semi-quantitative technique. **Table S2.** Determination of the sensitivity and specificity of the quantitative technique. **Table S3.** Determination of the sensitivity and specificity of PFGE. Click here for file
