# Supplementary material for: Diagnostic accuracy of semi-quantitative and quantitative culture techniques for the diagnosis of catheter-related infections in newborns and molecular typing of isolated microorganisms
Source: BMC Infect Dis. 2014 May 22;14:283. doi: 10.1186/1471-2334-14-283 (PMC4051137; doi:10.1186/1471-2334-14-283)
Supplement: Additional file 1: Table S1 — Determination of the sensitivity and specificity of the semi-quantitative technique. Table S2. Determination of the sensitivity and specificity of the quantitative technique. Table S3. Determination of the sensitivity and specificity of PFGE. [file 1471-2334-14-283-S1.docx]

**File name: Additional file 1**

Table S1. Determination of the sensitivity and specificity of the semi-quantitative technique

| Gold standard  (CR-BSI) | Semi-quantitative | | Total |
| --- | --- | --- | --- |
|  | Positive | Negative |  |
| Positive | 22 | 6 | 28 |
| Negative | 1 | 16 | 17 |
| Total | 23 | 22 | 45 |

Table S2. Determination of the sensitivity and specificity of the quantitative technique

| Gold standard  (CR-BSI) | Quantitative | | Total |
| --- | --- | --- | --- |
|  | Positive | Negative |  |
| Positive | 17 | 11 | 28 |
| Negative | 1 | 16 | 17 |
| Total | 18 | 27 | 45 |

Table S3. Determination of the sensitivity and specificity of PFGE

| Gold standard  (CR-BSI) | PFGE | | Total |
| --- | --- | --- | --- |
|  | Positive | Negative |  |
| Positive | 21 | 4 | 25 |
| Negative | 0 | 19 | 19 |
| Total | 21 | 23 | 44 |
